# hACE2-Induced Allosteric Activation in SARS-CoV versus SARS-CoV-2 Spike Assemblies Revealed by Structural Dynamics

**DOI:** 10.1021/acsinfecdis.3c00010

**Published:** 2023-05-11

**Authors:** Chengbo Chen, Richard Zhu, Edgar A. Hodge, Marco A. Díaz-Salinas, Adam Nguyen, James B. Munro, Kelly K. Lee

**Affiliations:** † Department of Medicinal Chemistry, University of Washington, Seattle, Washington 98195, USA; ‡ Biological Physics Structure and Design Program, University of Washington, Seattle, Washington 98195, USA; § Department of Microbiology and Physiological Systems, University of Massachusetts Chan Medical School, Worcester, Massachusetts 01605, USA

**Keywords:** COVID, allostery, SARS-CoV-2, fusion protein, HDX-MS, protein dynamics

## Abstract

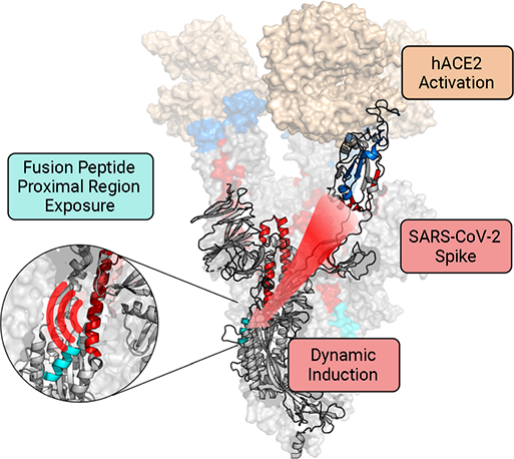

SARS-CoV and SARS-CoV-2 cell entry begins when spike glycoprotein (S) docks with the human ACE2 (hACE2) receptor. While the two coronaviruses share a common receptor and architecture of S, they exhibit differences in interactions with hACE2 as well as differences in proteolytic processing of S that trigger the fusion machine. Understanding how those differences impact S activation is key to understand its function and viral pathogenesis. Here, we investigate hACE2-induced activation in SARS-CoV and SARS-CoV-2 S using hydrogen/deuterium-exchange mass spectrometry (HDX-MS). HDX-MS revealed differences in dynamics in unbound S, including open/closed conformational switching and D614G-induced S stability. Upon hACE2 binding, notable differences in transduction of allosteric changes were observed extending from the receptor binding domain to regions proximal to proteolytic cleavage sites and the fusion peptide. Furthermore, we report that dimeric hACE2, the native oligomeric form of the receptor, does not lead to any more pronounced structural effect in S compared to saturated monomeric hACE2 binding. These experiments provide mechanistic insights into receptor-induced activation of *Sarbecovirus* spike proteins.

Coronaviruses have spilled over from animals into the human population at least three documented times in the past 20 years. Two of these involved viruses that are now classified in the *Sarbecovirus* subgenus, SARS-CoV (Severe Acute Respiratory Syndrome Coronavirus) and SARS-CoV-2. SARS-CoV, which gave rise to highly pathogenic infections, emerged in 2002 but was contained before it could spread widely across large populations.^1^ The first COVID-19 case, caused by the SARS-CoV-2 virus, was reported in December 2019.^2,3^ Compared with SARS-CoV, SARS-CoV-2 infection exhibits a longer incubation time, greater level of asymptomatic transmission, and overall higher transmissibility.^4^ SARS-CoV-2 was not contained, and once it began spreading widely in the human population, subsequent waves of variants emerged with mutations that led to enhanced transmissibility and altered antigenic profiles. To date, roughly 760 million infections and 6.8 million deaths have been documented as of March 2023 (WHO).

Both SARS-CoV and SARS-CoV-2 utilize trimeric spike glycoprotein (S) assemblies to recognize the hACE2 receptor on host cells.^5,6^ Each S protomer consists of a receptor binding subunit (S1) and a fusion subunit (S2), separated by a furin protease cleavage site. Prior to hACE2 binding, the cellular furin protease has a different activity on these two S glycoproteins. In SARS-CoV S, furin is ineffective in cleaving after the single arginine residue at position 667 (Figure 1a). In SARS-CoV-2, furin activity is enhanced by the polybasic RRAR motif, resulting in efficient cleavage of S into S1 and S2 subunits, which has been linked to enhanced pathogenicity in this virus (Figure 1a).^7−9^

**Figure 1:**
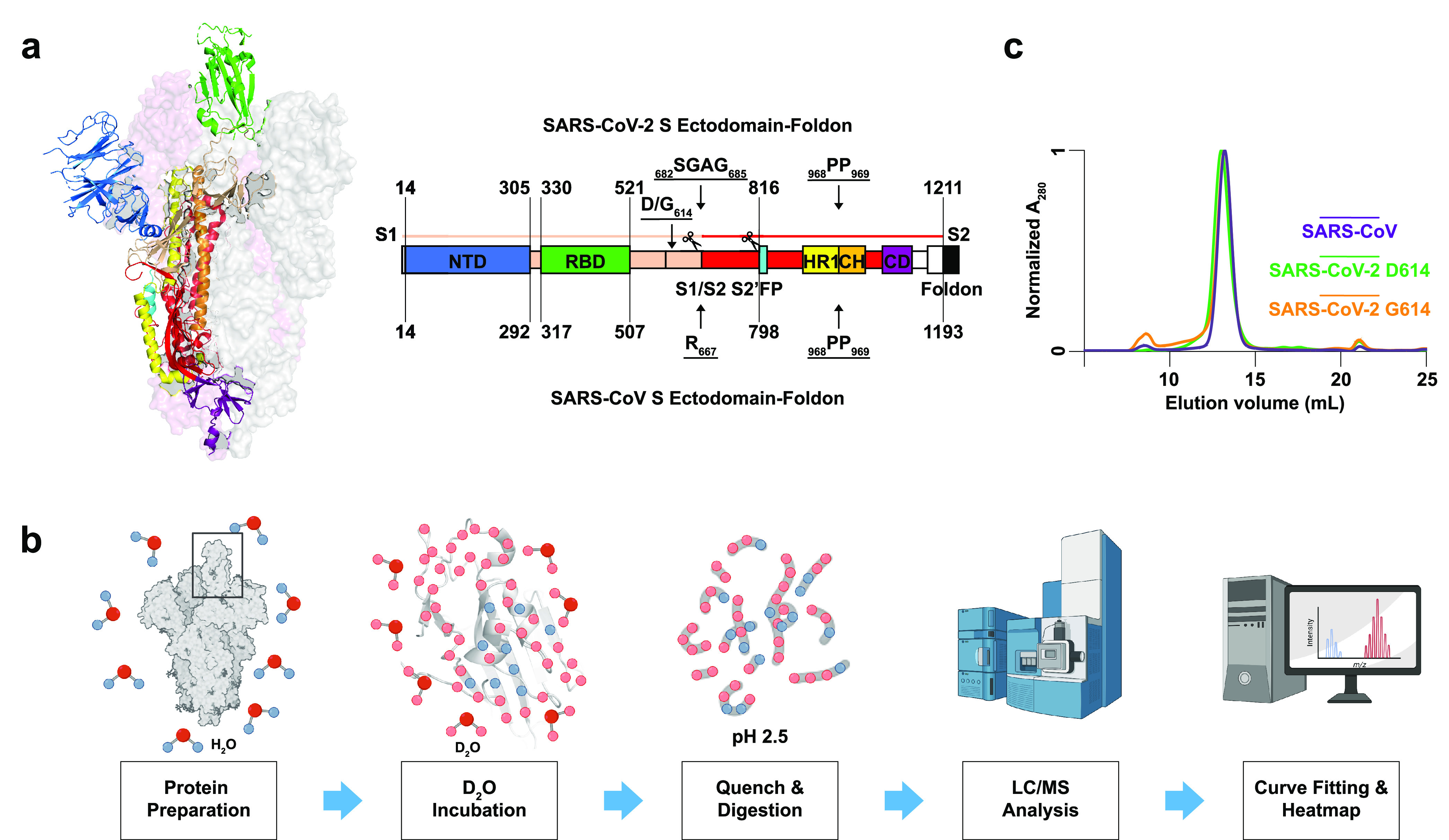
**Overview of HDX-MS experiments on CoV spikes.** (a) Structural organization of SARS-CoV-2 S ectodomain (PDB: 6VSB) and colored domain organization annotated with domain boundaries and mutation sites of SARS-CoV-2 S (upper) and SARS-CoV S (lower) ectodomains. S1: receptor binding subunit; S2: fusion subunit; NTD: N-terminal domain; RBD: receptor binding domain; FP: fusion peptide; HR1: heptad repeat 1; CH: central helix; CD: connector domain. (b) Schematic illustration of HDX-MS experiments. (c) Size-exclusion chromatography (SEC) trace for purified SARS-CoV, SARS-CoV-2 D614, and SARS-CoV-2 G614 S.

A multitude of soluble pre-fusion S ectodomain trimer cryo-EM structures of both SARS-CoV S and SARS-CoV-2 S have been reported in unbound as well as hACE2- and antibody-bound forms.^10−14^ These studies have determined the architecture of S assemblies across different strains, revealing open and closed conformations as well as resolving the detailed contacts between antibodies and hACE2 receptor with S. Successful binding of S in the open conformation to hACE2 is believed to prime the S cell entry machinery, inducing conformational changes that render distal regions at the host protease cleavage site (S2′) more susceptible to cleavage by TMPRSS2 and cathepsin, which free the fusion peptide (FP) and trigger refolding of the S2 subunit, leading to membrane fusion (Figure 1a).^15−19^ Once primed and triggered, like other Class I fusion proteins, S undergoes a dramatic refolding from pre-fusion to post-fusion conformation in order to complete the membrane fusion process.^20^

To go beyond static structures and better understand the mechanisms of spike priming and activation, techniques characterizing solution-state protein dynamics are critical. Single-molecule Förster resonance energy transfer (smFRET) studies using fluorescently labeled spikes have tracked the interconversion between receptor binding domain (RBD) up and down conformations and monitored the influence of antibody or hACE2 binding on this conformational landscape.^21−23^ Structural information from smFRET, however, is limited to the relative positioning and orientation of fluorescent probes that are introduced into the protein; thus, it is of interest to use complementary methods that enable us to investigate dynamics throughout complex viral glycoprotein assemblies. Hydrogen/deuterium-exchange mass spectrometry (HDX-MS) is a powerful approach for monitoring local protein structural dynamics under native solution conditions (Figure 1b). By tracking deuterium uptake kinetics for peptide segments throughout a given protein, HDX-MS can be particularly informative for identifying conformational changes resulting from mutations or ligand binding.^24,25^

To first investigate whether the similar spike architecture observed in two distantly related CoV S assemblies results in conserved dynamic profiles despite significant sequence differences, we used HDX-MS to profile local structural dynamics throughout the SARS-CoV and two SARS-CoV-2 S ectodomain trimers, one with glycine at residue 614 (G614) and one with aspartate (D614). The S-2P forms of these trimers, which include a di-proline substitution between HR1 and CH domains, were expressed in Expi293F cells to provide native glycosylation and folding of the S assemblies. The SARS-CoV-2 S constructs replaced the native polybasic RRAR S1/S2 cleavage site sequence with a SGAG linker sequence to help maintain the trimers in native, pre-fusion conformations (Figure 1a, Figure S1).^21^ The SARS-CoV S construct retained the native R, which is not cleaved under typical cell culture expression conditions (Figure S1). In all other respects, such engineered spike trimers have been observed to recapitulate the same native structure, antigenicity, immunogenicity, and ability to engage hACE2 as native, full-length, cleaved forms of the trimer.^10,13^ Rather than focusing on late SARS-CoV-2 variants of concern, we sought to compare the forms of the *Sarbecoviruses* that were closest to the spillover events, before significant adaptation to the new human host in the form of enhanced hACE2 interaction and evolution in response to immune selective pressure reshaped the structural profile and function of S. While hACE2 interactions are relatively weak in these founding viruses compared to some later SARS-CoV-2 variants, they were sufficient to enable infection of human cells.

Purification of the spikes yielded a single peak from size-exclusion chromatography (SEC) (Figure 1c). Dynamic light scattering (DLS), SDS-PAGE, and native PAGE confirmed the homogeneity of the trimeric S assemblies (Figure S1). HDX-MS experiments were performed on all three S constructs with incubation times ranging from 3 seconds to 20 hours. Tandem MS/MS analysis using a high-resolution Fusion Orbitrap mass spectrometer with an electron-transfer/higher-energy collision dissociation (EThcD) method, along with the aid of previously reported glycan profiling,^26−28^ yielded high sequence coverage for SARS-CoV (70%, 9 N-glycosylation sites) and SARS-CoV-2 G614 and D614 (85%, 11 N-glycosylation sites) S constructs (Figure S2).

HDX-MS analysis revealed similarities and differences in the dynamic profiles of pre-fusion S in SARS-CoV and SARS-CoV-2 D614 and G614 trimers, as summarized in HDX-MS heatmaps (Figure S3) and corresponding butterfly plots showing deuteration trends at all four timepoints across the spike sequences (Figure 2a). The overall deuteration trends in SARS-CoV and SARS-CoV-2 S trimers showed mostly similar domain-level trends across the proteins’ sequences. Only ∼76% sequence homology exists between SARS-CoV S and SARS-CoV-2 S. As a result, enzymatic digestion patterns differed to a significant extent, and the number of peptides with the same number of exchangeable amides that could be quantitatively compared was somewhat limited.^3,11,29^

**Figure 2:**
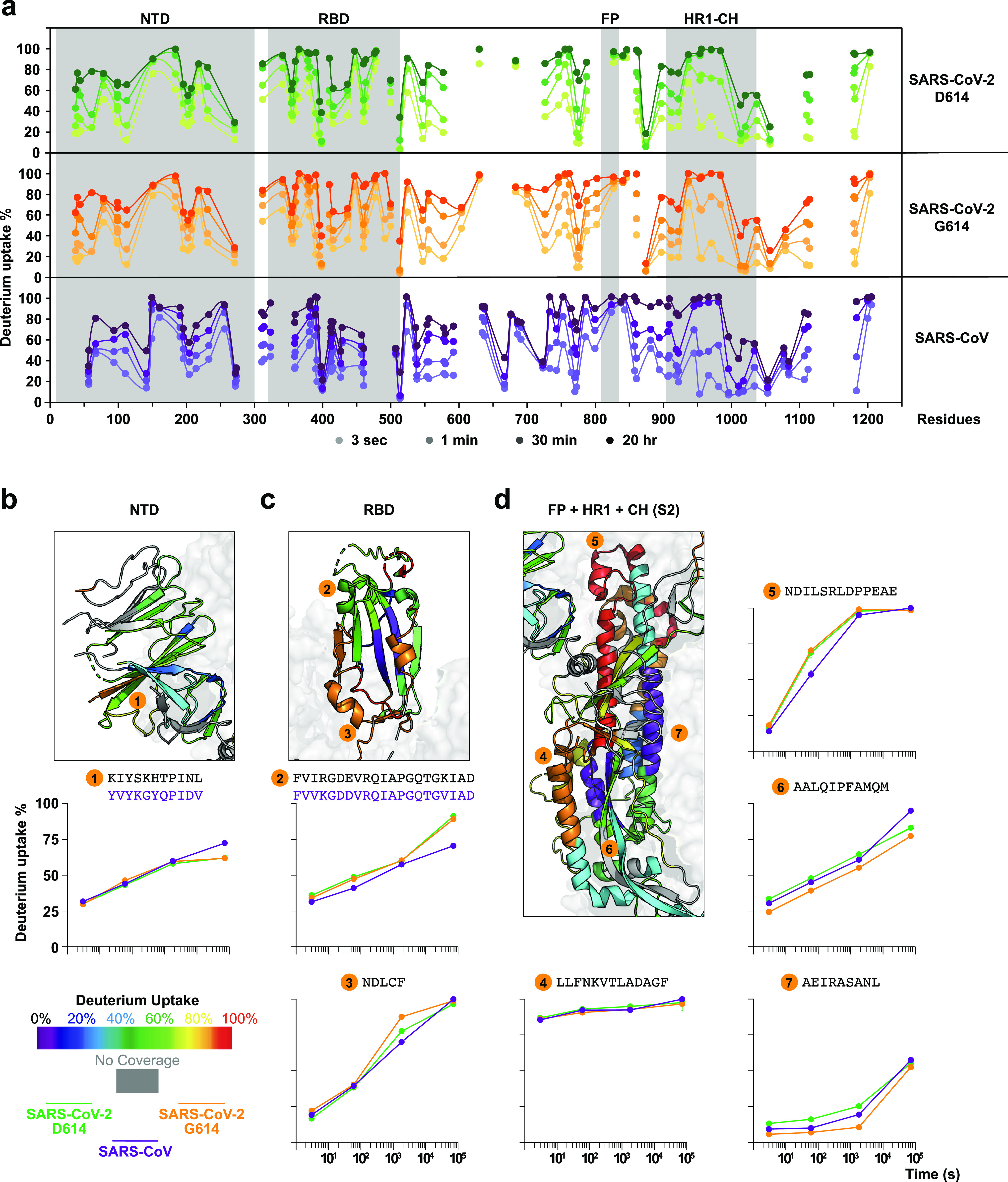
**HDX-MS reveals structural dynamic differences in SARS-CoV versus SARS-CoV-2 S trimers.** (a) Butterfly plots of SARS-CoV-2 D614, SARS-CoV-2 G614, and SARS-CoV S reveal the similarities and differences in dynamic behavior across the spike sequences. Detailed comparisons of gray shaded regions for the (b) NTD, (c) RBD, and (d) S2 subunit are presented with deuterium uptake heatmaps of SARS-CoV-2 G614 S at 30-min exchange timepoints and numbered uptake plots between homologous peptide sequences from SARS-CoV S versus two forms of SARS-CoV-2 S glycoproteins.

For the available homologous peptides, quantitative and direct comparisons revealed measurable differences in dynamics among S trimers (Figure 2b–d). Homologous peptides in NTD in SARS-CoV S were observed to be similar in the early to middle exchange time frame but more dynamic than in SARS-CoV-2 S at later timepoints (Figure 2b). In the meantime, homologous peptides in RBD including part of the receptor binding motif (RBM) and C-terminal region indicated greater local dynamics in SARS-CoV-2 S (Figure 2c). Both of these differences could be a consequence of SARS-CoV-2 S adopting more open conformations than SARS-CoV S, as the accessibility of these peptides is affected by the RBD up/down positioning based upon reported structures.^12,30^ A similar effect was also reflected by higher dynamics at the top of the internal helical bundle in the SARS-CoV-2 S (Figure 2d, peptide #5). Highly dynamic fusion peptide regions (Figure 2d, peptide #4) were observed in all S trimers. Our findings reinforce the cryo-EM-based observation that SARS-CoV-2 S favors more open conformations than SARS-CoV S, while the overall architecture is relatively conserved at a structural dynamic level, despite the significant sequence differences.^30−33^

The D614G mutation that emerged from the ancestral Wuhan SARS-CoV-2 strain was the first pivotal mutation that appeared as SARS-CoV-2 adapted to increase transmission through the human population.^34^ Residue 614 is positioned at a central site at the interface of S1 and S2 subunits (Figure 1a, Figure S4), and cryo-EM structures revealed that the D614G mutation bolstered contacts between the two subunits.^31^ The HDX-MS butterfly and difference plots comparing SARS-CoV-2 spikes (Figure 2a, Figure S4) revealed a surprising degree of differences resulting from the single residue substitution, larger in many cases than the differences between SARS-CoV-2 G614 and SARS-CoV S dynamics, which are far more divergent in sequence. Two regions, the hinge about which the RBD pivots relative to the rest of the S1 subunit as well as much of the S2 subunit, exhibited substantial dynamic differences between the two SARS-CoV-2 S assemblies (Figure 2c,d, Figure S4). The increased deuteration, indicative of greater solvent accessibility, across the RBD peptides (Figure 2c, peptide #3) in the G614 mutant is in agreement with cryo-EM data that suggested the D614G mutation shifted the dynamic equilibrium toward an RBD-up state.^30,32^ The D614G mutation also led to greater ordering of the S2 base of the trimer at sites extending well beyond the local interface around the mutation highlighted by cryo-EM imaging of G614 versus D614 S-2P trimers (Figure 2d, peptides #6 and #7, Figure S4).^30,31^ Notably, peptides at the bottom of the heptad repeat 1 (HR1) and central helix (CH) domains in the ancestral D614 S exhibited greater levels of deuterium uptake and greater dynamics while also exhibiting bimodal mass spectral envelopes, indicating that this region samples more than one conformational state (Figure S5).

The G614 spike by contrast appeared more ordered and conformationally homogeneous, and it retained the protected peptide backbone at these sites. These differences in local dynamics are consistent with global stability measured by monitoring the DLS signal over the course of thermal denaturation experiments (Figure S1). While the D614 trimer began to undergo thermal denaturation leading to aggregation and an increase in turbidity around 50 °C, the G614 trimer maintained a constant radius of hydration until above 60 °C when it started to show a modest increase in radius by scattering.^35^ These data indicate that the D614G mutation conferred a significant increase in spike stability. Residue 614 and surrounding areas at the interface of S1 and S2 may serve as a key linchpin that mediates trimer integrity^31^ and S2’s dynamic phenotype, which can impact the spike’s propensity to triggering and activation.

The comparison of SARS-CoV-2 D614 and G614 S assemblies indicated long-range dynamic coupling between RBD in the spike apex and key functional regions in S2 and showed good agreement with two recent studies.^36,37^ We next sought to investigate how spike activation from the hACE2–RBD interaction impacts dynamics in the S2 subunit as well as its contribution to viral fusion. In this case, due to the relatively lower stability in SARS-CoV-2 D614 S and difficulty in enriching stable trimer at the desired concentration for further studies, G614 spike was used to investigate how the SARS-CoV-2 S assembly responds to hACE2 activation in comparison with SARS-CoV S. We incubated soluble monomeric hACE2 with the SARS-CoV S trimer, SARS-CoV-2 G614 S trimer, and trimeric tethered SARS-CoV-2 RBD (as an all-RBD-up control) at a molar ratio of 9:1 (3-fold molar excess per protomer), reaching a >99% bound fraction based on their *K*_D_ values.^10,38^ Furthermore, we compared the effect of dimeric hACE2 with monomeric hACE2 on S conformation in order to test the hypothesis that the potential for bivalent engagement of the trimer may enhance its dynamic activation, which has been suggested in other reports but not investigated using experimental approaches.^39^

SARS-CoV-2 RBD domains tethered by a flexible linker to a trimerization motif served as a simplified mimic of spike with an all-RBD-up conformation exposing all three RBMs to the hACE2.^28^ Local protection induced by hACE2 binding was observed by HDX-MS across the RBM (Figure 3a) and extended down to the β-strand core (Figure S6). Conversely, peptides distal to the RBM did not change in amide exposure levels following hACE2 binding (Figure 3a, Figure S6).

**Figure 3:**
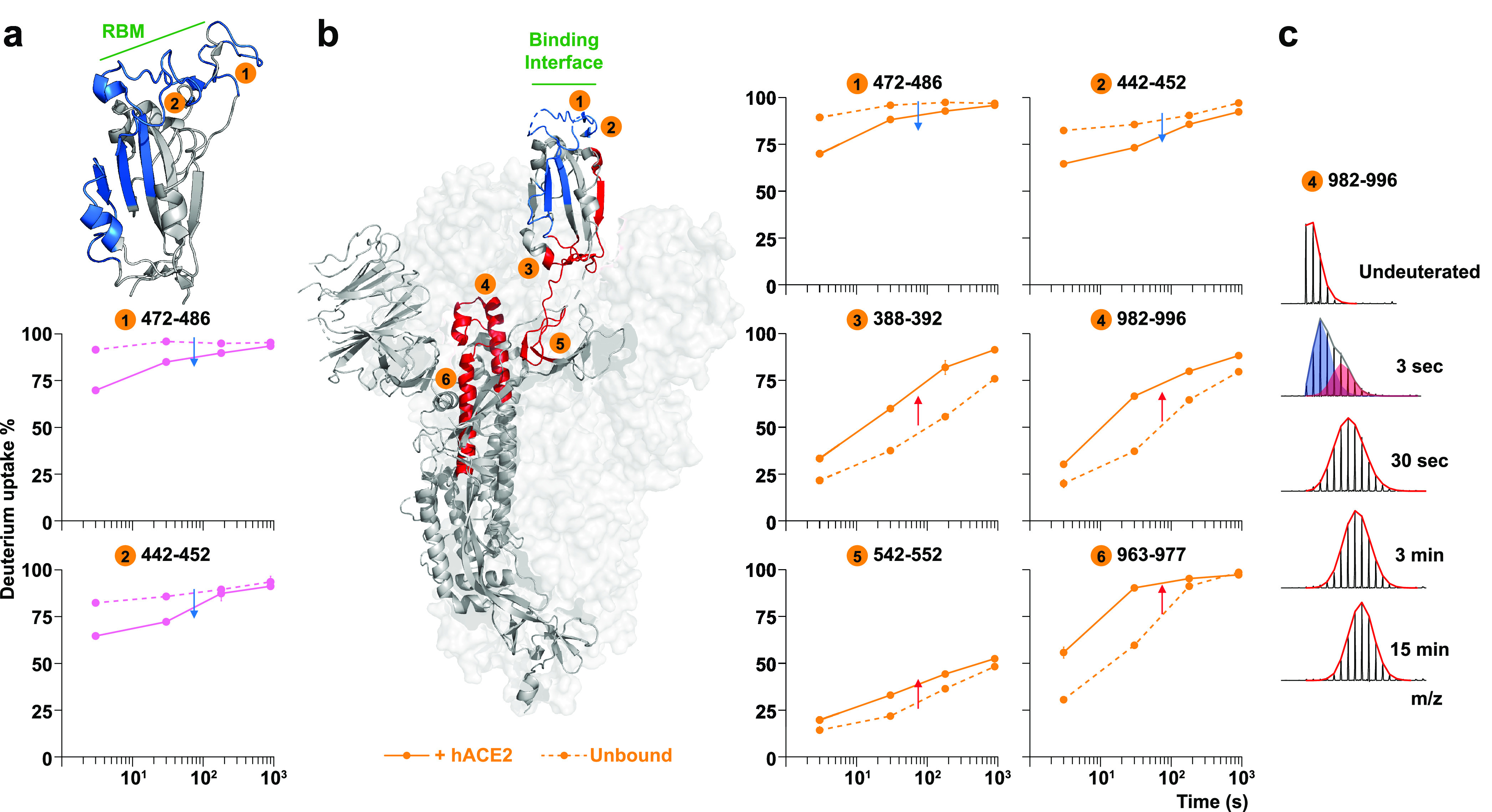
**Local and allosteric effects on SARS-CoV-2 trimeric tethered RBD and trimeric S upon hACE2 binding.** (a) Only local protection has been observed on the trimeric tethered RBD construct (PDB: 6W41). Blue indicates more protection and red indicates more exposure after hACE2 binding. (b) On hACE2-bound S trimer, the corresponding RBD shows protection throughout the RBM (peptides #1 and #2) but more exposure at the C terminal (#3); the hinge region shows increased dynamics (#5), and the central helices show increased accessibility from the fusion peptide proximal region (#6) to the helical apex (#4). (c) A region in the central helices’ apex (#4 in panel b) shows a bimodal mass envelope at the 3-s deuteration timepoint. The bimodal spectrum was binomially fit to two populations: a protected population is shaded blue, and the heavier in mass population that has sampled an exposed conformation is shaded red.

In the context of SARS-CoV-2 G614 S, the RBD region responded to hACE2 binding with similar local protection as in isolated RBD, leading to the β-strand core (Figure 3b, peptides #1 and #2). However, greater exposure and deuterium exchange occurred for peptides 388–392 at the RBD hinge site in hACE2-bound S trimers (Figure 3b, peptide #3), indicating that the conformational bias of RBDs on S was shifted to a state consistent with an “up” conformation. C-terminal peptides in spike RBD showed greater protection and reduced deuteration than corresponding peptides on tethered RBD due to the interaction and connections to the rest of the spike structure (Figure 3b, peptide #3, Figure S6).

Additionally, hACE2 binding led to enhanced dynamic behavior in broad regions of the spike, including allosteric changes at sites distant from the RBD (Figure 3b, Figure S7). Peptides in the NTD responded with only modest changes (Figure S7), while more prominent allosteric changes extended to the hinge region (Figure 3b, peptide #5) and to the top of the central helical bundle of the S2 subunit from HR1 to the CH domain (Figure 3b, peptides #4 and #6). A bimodal spectrum was observed in the peptide #4 with two co-existing populations exhibiting differences in protection at the earliest timepoint, suggestive of conformational switching between two states (Figure 3c). hACE2 binding also destabilized the HR1 helix as observed in the uptake plot where this region became nearly fully exchanged after 30 s (Figure 3b, peptide #6, Figure S7). The flexibility of this central helix apex in the fusion machinery is consistent with a distorted helical structure observed in cryo-EM structures when hACE2 was bound.^40^ Even more extreme forms of distorted, splayed open trimers have been reported whose formation was promoted by hACE2 binding.^41^ We did not observe evidence of such splayed open trimers, though we did observe peptides with fast exchange rates and bimodal spectra resolved at multiple regions across HR1 and CH domains, indicating transient conformational sampling of these relatively dynamic regions (Figure S7).

Peptides revealing increased dynamics resulting from hACE2 binding were also identified in the fusion peptide proximal region (FPPR), which is adjacent to the TMPRSS2 cleavage site (Figure 3b, peptide #6, Figure S7). We conclude that the receptor-induced increases in dynamics in the SARS-CoV-2 G614 S at the FPPR site likely results in a primed conformation with enhanced accessibility for TMPRSS2, which must cleave the S2′ site in order to free the fusion peptide and trigger the spike’s fusion activity.

In comparison, we also examined hACE2’s impact on SARS-CoV S. hACE2 induced dynamic changes in the SARS-CoV S trimer with both direct protection in the RBM (Figure 4a, peptide #1) and distal allosteric changes resulting in increased exposure at the C-terminal segment of the RBD (#2), the hinge region connecting the C-terminal of the RBD (#3), the apex of the CH (#4), and the HR1 helix (#5 and #6) (Figure 4a, Figure S8). hACE2 binding induced the RBD to adopt primarily an up conformation as reflected by the peptide near the RBD C-terminus (NDLCF, residues 375–379) exhibiting increased deuterium exchange. A bimodal spectrum was resolvable at the binding interface (Figure 4b, corresponding peptide #1 in Figure 4a). In the unbound state, peptide #1’s deuterium uptake plateaued at 50%, indicating that half of the peptide residues at the binding interface were highly exposed and fully deuterated at an early timepoint while the other half were barely deuterated, being highly protected in the RBD β-strand core. This likely results from the tendency for hACE2 to dissociate as reflected by the fast *k*_off_ for the hACE2-SARS-CoV spike complex.^10^ The two populations in the mass spectra reflect an hACE2-bound and an unbound population, where the protected population in the bimodal spectrum at the 3-s timepoint quickly transitioned to the exposed, more deuterated population when the receptor dissociated.

**Figure 4:**
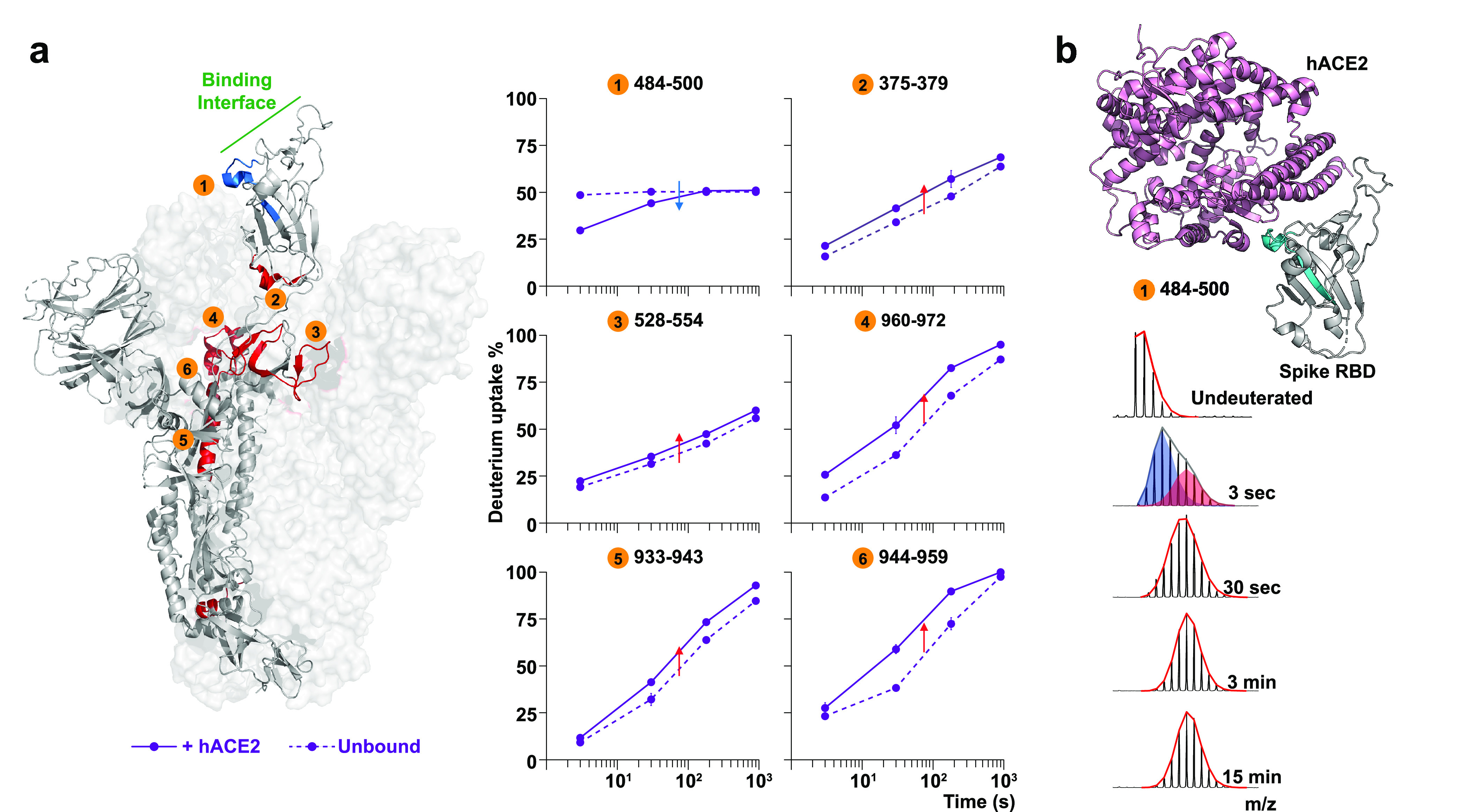
**Local and allosteric effects on SARS-CoV S upon hACE2 binding.** (a) Upon hACE2 binding, the RBD shows protection across the binding interface (peptide #1) and increased exposure at the C terminus (#2); the hinge region shows increased dynamics (#3), and the central helices reveal increased amide accessibility (#4–#6). Blue indicates more protection and red indicates more exposure after hACE2 binding (PDB: 6CRZ). (b) A region at the binding interface exhibits a bimodal mass envelope at the 3-s timepoint (PDB: 3D0G). The bimodal spectrum was binomially fit to two populations: a protected population is shaded blue, and the heavier in mass population that has sampled an exposed conformation is shaded red.

The similarities and differences in hACE2-induced dynamic changes in SARS-CoV and SARS-CoV-2 G614 S are shown in parallel in Figure 5a,b. Binding of hACE2 to both SARS-CoV and SARS-CoV-2 G614 S revealed a notable allosteric effect extending from the RBD at the spike apex all the way down the central helices in the S2 subunit (Figure 5a, Figure 5b). These dynamic changes in SARS-CoV-2 G614 S were transduced throughout the trimer in a more widespread fashion, especially seen in ordering of the RBD, due to the SARS-CoV-2 spike’s more stable binding to hACE2, and along the S2 subunit’s central helical region (HR1-CH) at the early exchange timepoints. In SARS-CoV S, the effects were more muted, perhaps due to the greater likelihood of hACE2 to be dissociated from this S, resulting in a reduced propensity for the S2 subunit to become activated.

**Figure 5:**
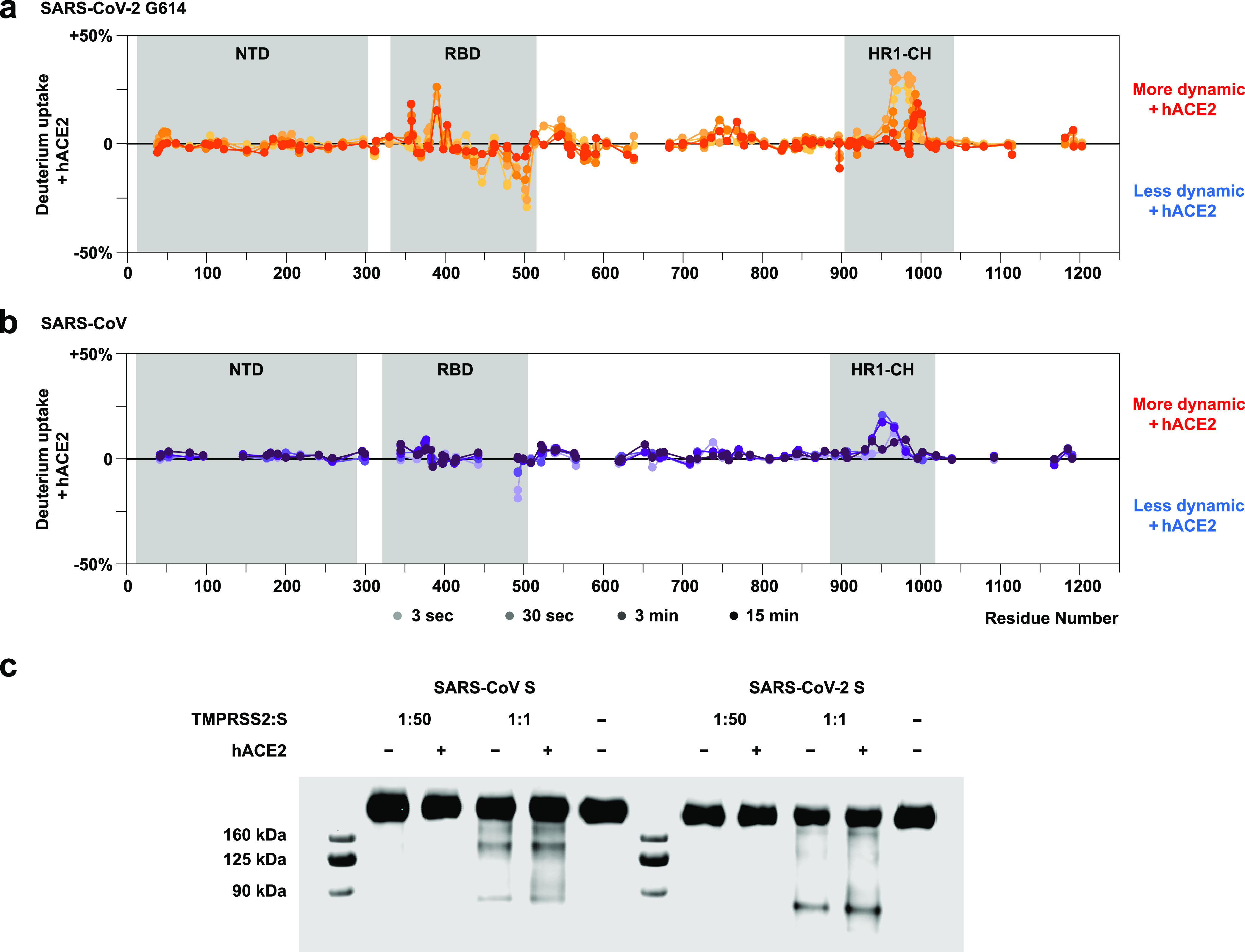
**Dynamic impacts on S from hACE2 binding and subsequent effect on TMPRSS2 digestion.** Difference butterfly plots of (a) SARS-CoV-2 S G614 and (b) SARS-CoV S indicate variations in footprints and allosteric effects resulting from hACE2 binding. Gray shadings highlight the RBD, fusion peptide, and HR1-CH region. Higher and more widespread dynamics induction is observed over the S2 central helical region (HR1-CH) of SARS-CoV-2 S than SARS-CoV S at the early exchange timepoints. (c) Western blot indicating TMPRSS2 digests more efficiently on SARS-CoV-2 S. The occurrence of stable digested product at ∼90 kDa is enhanced with hACE2 binding.

hACE2 exists as a dimer when displayed on the cell surface, and it has been proposed that S, which displays three RBDs and native, dimeric hACE2, may exhibit avid interactions resulting from the receptor’s bivalency.^42^ Here, we sought to determine whether the ability of dimeric hACE2 to interact bivalently with more than one RBD in S trimers potentially leads to a greater degree of conformational priming of the spike compared to monomeric hACE2 binding. Parallel HDX-MS experiments performed with dimeric and monomeric hACE2 bound to SARS-CoV-2 S at pH 7.4 showed nearly superimposable changes in structural dynamics for all peptides that exhibit prominent differences in response to hACE2 binding (Figure S9). Bivalent presentation of the S-interactive domains on hACE2 thus appears to not facilitate engagement with multiple RBDs on S. It is possible that the reported effects of hACE2 bivalency may thus involve inter-spike cross-linking on viral particles rather than bivalent intra-spike RBD engagement.^22^

A complementary digestion assay was performed to investigate the hACE2 binding effect on subsequent accessibility of protease TMPRSS2 to S in solution (Figure 5c). Western blots probing with a primary antibody against the RBD showed undigested S bands above 160 kDa and a digested product peptide band at ∼90 kDa when TMPRSS2 was added in a 1:1 ratio to S for both SARS-CoV and SARS-CoV-2 S. The 90 kDa RBD-containing peptide reflects a proteolytic product generated by TMPRSS2 cleavage at or near the S2′ position (Figure S10). It is further confirmed to be a peptide from the C-terminal of the RBD to the S2′ site by comparing the results from the non-reducing condition and a primary antibody capable of blotting the N-terminal of S2 prior to the S2′ site (Figure S10), consistent with the known biological activity of TMPRSS2 and previous studies.^43,44^ Even in the absence of receptor binding, a faint digest product was observed for both SARS-CoV and SARS-CoV-2 S. SARS-CoV-2 S showed a greater extent of digest product, and in both cases hACE2 binding enhanced TMPRSS2 digestion (Figure 5c), with enhancement in cleavage at the C-terminal to the RBD being consistent with the S2′ position where allosteric structural dynamic changes were detected in SARS-CoV-2 S from HDX-MS (Figure 3b). These results support the hypothesis that hACE2 binding induces allosteric conformational changes that extend to the S2′ cleavage site that prime the spike for TMPRSS2 cleavage activation.

Class I fusion proteins are dynamic macromolecules that undergo dramatic conformational changes during the viral entry process. Their intrinsic dynamic properties are anticipated to impact how they engage with host receptors as well as their activation, resulting in differences in fusogenicity and infectivity. Here, using HDX-MS, we identified notable differences in the structural dynamics between *Sarbecovirus* pre-fusion S assemblies. The results align with the fact that prevalent G614 strain with higher transmissibility showed more preferred open conformations in the RBD crown, balanced by increased stabilization in the S1/S2 contact sites in the spike’s base. These findings are consistent with and complement previous cryo-EM data showing a more open conformation in the G614 S trimer.^21,30,32,33^ Similarly, in a smFRET study of S, the D614G mutation also resulted in S shifting to a more open conformation.^23^ We infer that the bias toward an open state, which by itself could be destabilizing, is compensated by the increased ordering of the S2 subunit, thus resulting in an evolutionarily advantageous spike configuration that facilitates hACE2 engagement while maintaining spike integrity.

Other HDX-MS studies have sought to fill the gap between static structural data and biological functions through investigation of protein dynamics. For example, an HDX-MS and molecular dynamic (MD) simulation study of the ancestral Wuhan D614 spike first described an allosteric effect with an increase in structural order at the S1/S2 cleavage site resulting from hACE2 binding.^45^ Interestingly, our observation from G614 S showed this loop at the S1/S2 site exhibited similar, high levels of flexibility with and without hACE2 binding. Furthermore, we observed a receptor-induced allosteric effect that destabilized the fusion peptide proximal region of SARS-CoV-2 S (Figure 3b). A recent study using HDX-MS to study a range of SARS-CoV-2 variants likewise noted hACE2 induced effects in the FPPR,^37^ consistent with our observations, which we further investigated by TMPRSS2 cleavage assays. Our current study extends dynamic investigations of the *Sarbecovirus* beyond the SARS-CoV-2 family by examining the behavior and receptor-induced activation of SARS-CoV as well. This comparison is of interest due to the common hACE2 receptor usage and significant sequence variation between the S fusion proteins as well as the differences in hACE2-RBD interactions in the two *Sarbecovirus* cases.

The hACE2 activation pathway we have examined is informative in reflecting the priming of the spike for the subsequent cleavage at the S2′ position, by either TMPRSS2 or cathepsin proteases, which is required to free the fusion peptide and trigger the subsequent conformational changes required for membrane fusion. One potential reason that increased dynamics in the FPPR were observed in SARS-CoV-2 S but not SARS-CoV S might be the differences in how the spikes interact with hACE2 (Figure 5). Biolayer interferometry data^10^ indicates that hACE2 dissociates more slowly from SARS-CoV-2 S than SARS-CoV S. Tighter hACE2 binding may facilitate greater dynamic induction and increased FPPR exposure in SARS-CoV-2 that is required for TMPRSS2’s access to the S2′ cleavage site and subsequent fusion triggering. In this respect, SARS-CoV likely requires greater saturation of binding sites and possibly greater spike avidity on the virion surface with membrane-bound hACE2 in order to be primed to a similar extent.

These results reveal key allosteric pathways as well as virus and variant-specific differences in dynamic activation among *Sarbecovirus* S assemblies. Having such an understanding complements high-resolution structural information and provides mechanistic insight into differences in viral infectivity and function during cell entry.

## Methods

### Plasmid Construction

The gene sequences coding the ectodomains of SARS-CoV S (residues 14–1190) and SARS-CoV-2 S (residues 14–1211) with di-proline mutations (_968_KV_969_ in SARS-CoV and _986_KV_987_ in SARS-CoV-2), SGAG substitution at the S1/S2 site (_682_RRAR_685_ in SARS-CoV-2) and C-terminal T4 fibritin foldon, a TEV protease cleavage site, and His-tag were codon-optimized and synthesized by GenScript, then cloned into pcDNA3.1(−). D614G mutation on SARS-CoV-2 S was performed with a Q5 Site-Directed Mutagenesis Kit (NEB). Plasmids for soluble monomeric and dimeric hACE2 with His-tag were purchased from Addgene (plasmids #149268 and #154101). Open reading frame sequences were confirmed using Sanger sequencing (Genewiz).

### Transient Transfection

Expi293F cultures were transfected at ∼3 × 10^6^ cells/mL with plasmid DNA stated above, respectively. For each liter of culture, 1 mg DNA and 3 mg polyethylenimine (PEI) were separately diluted with 25 mL Gibco Opti-MEM (Thermo Fisher Scientific), incubated for 5 min, and then thoroughly mixed. Each transfection mixture was allowed to stand for 15 min and then added dropwise into the culture with gentle swirling. The transfected cultures were incubated at 37 °C and 8% CO_2_ and shaken at 125 RPM.

### Protein Purification

Transfected cultures were harvested on the fifth day post-transfection (viability ∼40%) by centrifugation at 2000 RCF for 20 min. Supernatants were vacuum-filtered via 0.45-μm αPES filters. Tris hydrochloride (Tris-HCl, pH 8.0) and arginine hydrochloride (ArgCl, pH 6.5) were supplemented to the filtered supernatant to a final concentration of 10 mM Tris-HCl and 50 mM ArgCl as stabilizing excipient.^46^ A 5 mL Ni-NTA resin per liter supernatant was washed and equilibrated with Tris-ArgCl buffer (50 mM Tris-HCl pH 8.0, 100 mM ArgCl, 150 mM NaCl) before adding to supernatant for the batch binding at room temperature for 2 h. The mixture was then passed through reusable gravity columns (Bio-Rad) with the flow-through being collected. The resin was washed three times with three column volumes of wash buffer (Tris-ArgCl buffer with 5 mM imidazole). The bound protein was then eluted with one column volume of elution buffer (Tris-ArgCl buffer with 500 mM imidazole) three times. SDS-PAGE-checked elution fractions were combined and buffer-exchanged to Tris-ArgCl buffer containing 0.02% sodium azide (NaN_3_), then concentrated to less than 500 μL with an Amicon Ultra-4-ml centrifugal filter (30 K, Millipore) prior to injection. SEC purification was conducted on a Superose 6 10/300 GL column (Cytiva) on an AKTA Pure (GE) system. With the Tris-ArgCl running buffer and flow rate at 0.5 mL/min, the spike trimer was purified and eluted at around 14 mL (Figure 1d) while monomeric hACE2 was eluted at around 16 mL. The purified proteins were concentrated to 1 mg/mL, flash frozen in liquid nitrogen, and kept in −80 °C for future experiments.

### Hydrogen/Deuterium-Exchange Mass Spectrometry

For the experiment comparing SARS-CoV, SARS-CoV-2 D614, and SARS-CoV-2 G614 soluble spike dynamics, 10 μg of each sample was incubated in the deuteration buffer (10 mM Tris, pH* 8.0, 85% D_2_O, Cambridge Isotope Laboratories, Inc.) at 23 °C for four different timepoints: 3, 60, 1800, and 72 000 s. For the experiments featuring hACE2 binding impacts, 10 μg of SARS-CoV S, 10 μg of SARS-CoV-2 G614 S, and 3 μg of trimeric tethered SARS-CoV-2 RBD were incubated and equilibrated with hACE2 with the molar ratio 1:9 (trimer:hACE2) overnight at 23 °C, respectively. Both hACE2-bound and unbound samples were then deuterium-exchanged in the same deuteration buffer described above at 23 °C for 3, 30, 180, and 900 s. All H/D-exchanged samples were immediately mixed with an equal volume of ice-cold quench buffer (8 M urea, 200 mM tris(2-chloroethyl) phosphate, 0.2% formic acid (FA)) to pH 2.5 and flash-frozen in liquid nitrogen. Samples were analyzed by LC-MS using a Synapt G2-Si mass spectrometer (Waters) with the settings described in our previous spike proteomics study.^28^ Quenched protein samples were digested into peptides from an immobilized pepsin column (2.1 × 50 mm) with loading buffer (2% acetonitrile (ACN), 0.1% trifluoroacetic acid (TFA)) at 400 μL/min. Peptides were trapped on a CSH C18 trap cartridge (Waters), followed by separation over a CSH C18 column (1 × 100 mm, 1.7 μm, Waters) with a 20-min linear gradient from 3% to 40% buffer B (buffer A: 2% ACN, 0.1% FA, 0.025% TFA; buffer B: 99.9% ACN, 0.1% FA) at a flow rate of 40 μL/min. The totally deuterated control was prepared by collecting pepsin-digested peptide elution, drying by speed-vacuum, and incubating in deuteration buffer for 1 h at 85 °C. The peptides were identified from DriftScope (Waters) and analyzed by HDExaminer (Sierra Analytics). The obtained spectra were binomially fitted and visualized with HX-Express v2 on Microsoft Excel.^47^

## Supporting Information Available

Additional methods, supplementary figures, and supplementary tables (PDF)
Excel sheets of processed MS data (XLSX)
