# Supplementary material for: hACE2-Induced Allosteric Activation in SARS-CoV versus SARS-CoV-2 Spike Assemblies Revealed by Structural Dynamics
Source: ACS Infect Dis. 2023 May 11;9(6):1180–9. doi: 10.1021/acsinfecdis.3c00010 (PMC10228703; doi:10.1021/acsinfecdis.3c00010)
Supplement: Supplementary file 1 — id3c00010_si_001.pdf [file id3c00010_si_001.pdf]

Supporting Information

hACE2-Induced Allosteric Activation in SARS-CoV versus SARS-CoV-2 Spike Assemblies Revealed by Structural Dynamics

Chengbo Chen,[1,2] Richard Zhu,[1] Edgar A Hodge,[1] Marco A Díaz-Salinas,[3] Adam Nguyen,[2,4] James B Munro,[3] Kelly K Lee[1,2,*]

[1] Department of Medicinal Chemistry, University of Washington, Seattle, WA 98195, USA

[2] Biological Physics Structure and Design Program, University of Washington, Seattle, WA 98195, USA

[3] Department of Microbiology and Physiological Systems, University of Massachusetts Chan Medical School, Worcester, MA 01605, USA

[4] Current address: Division of Basic Sciences, Fred Hutchinson Cancer Center, Seattle, WA 98109, USA

[*]Corresponding author

Kelly K Lee: kklee@uw.edu

This supplementary file includes:

Methods

Figures S1 to S10

Tables S1 to S3

Methods

Gel Electrophoresis

Both Native-PAGE and SDS-PAGE gels were used in characterizing spike trimers. The 4-12% Bis-Tris Native-PAGE gel (Invitrogen) was run to check SEC fractions. Each lane was loaded with 20 μL sample, consisting of 15 μL SEC fraction sample and 5 μL 4× Native-PAGE loading dye (Invitrogen). Gel was run with 1× Native-PAGE cathode buffer (Invitrogen) and 1× Native-PAGE running buffer (Invitrogen) inside the gel chamber and 1× Native-PAGE running buffer outside the gel chamber at 150 Volts for 2 hours on ice. For SDS-PAGE gel, protein samples were mixed with 4× SDS-PAGE loading dye and boiled for 5 minutes. MES SDS running buffer (Invitrogen) was used. For the imaging process, both Native-PAGE and SDS-PAGE gels were first rocked in fix buffer (50% methanol, 7% acetic acid) for 30 minutes to fix, followed by stained with Coomassie Blue for 10 minutes, then de-stained in wash buffer (10% methanol, 7% acetic acid) overnight. The gels were imaged on the Odyssey® M imaging system (LI-COR Biosciences).

Western Blot

Western blot was used to check the S peptides digested from TMPRSS2. Both SARS-CoV and SARS-CoV-2 S trimers were incubated with hACE2 or Bovine serum albumin (BSA, served as negative control) in the same ratio as HDX conditions. Soluble TMPRSS2 (Creative Biomart Inc.) was incubated with S in 1:50, 1:10, and 1:1 ratio (w/w), respectively, at room temperature for 10 minutes. The digested samples (~40 ng S) were run on the SDS-PAGE gel with Chameleon® duo pre-stained ladder (LI-COR Biosciences). The peptides were transferred to the Immobilon®-FL PVDF membrane (Millipore) using NuPAGE™ transfer buffer (Invitrogen) at 30 Volts for 2 hours. The membranes were then blocked in 5% non-fat milk overnight and blotted by primary antibody

against spike RBD (Rabbit pAb, Sino Biological 40592-T62) or antibody against spike S2 subunit (Rabbit pAb, Sino Biological 40590-T62) in 1:2000 tris-buffered saline-Tween (TBST) for an hour and secondary antibody anti-Rabbit IgG Alexa Fluor™ 680 (Invitrogen) in 1:10000 TBST for an hour in room temperature. The membranes were imaged on the Odyssey® M imaging system (LI-COR Biosciences) using 700 nm channel and exposure intensity at 3.

Dynamic Light Scattering

Dynamic light scattering (DLS) was used to characterize the trimer integrity and homogeneity by measuring hydrodynamic radius and polydispersity. SEC purified spike samples for DLS were first spun down at 25000 RCF for 20 minutes to remove aggregation pellet, if any. For each DLS run, 5 µL of spun-down DLS sample was drawn up from just beneath the meniscus of the sample and carefully injected into the flow cell (DynaPro NanoStar, Wyatt). Each run was measured with twenty 10-second acquisitions by auto-attenuation of the LASER.

Statistical Analysis

Two biological replicates were prepared for SARS-CoV S and SARS-CoV-2 G614 S from two separate protein purifications. One preparation was used to compare unliganded S dynamic behaviors together with SARS-CoV-2 D614 S for HDX at 3, 60, 1800 and 72000 seconds. The other preparation was used to compare SARS-CoV S and SARS-CoV-2 G614 S dynamic behaviors in response to hACE binding for HDX at 3, 30, 180 and 900 seconds. Two technical replicates were performed for each time point. There are in total four replicates for SARS-CoV S and SARS-CoV-2 G614 S at 3 second time point to compare reproducibility of the experimental HDX-MS system. Two-tailed t-test was used to report the statistics of SARS-CoV S and SARS-

CoV-2 G614 S uptake levels between unbound and hACE2-bound states. All uptake plots include error bars indicating the standard derivations from the replicates. Error bars less than ±2% were covered by the data points.

Figures and Tables

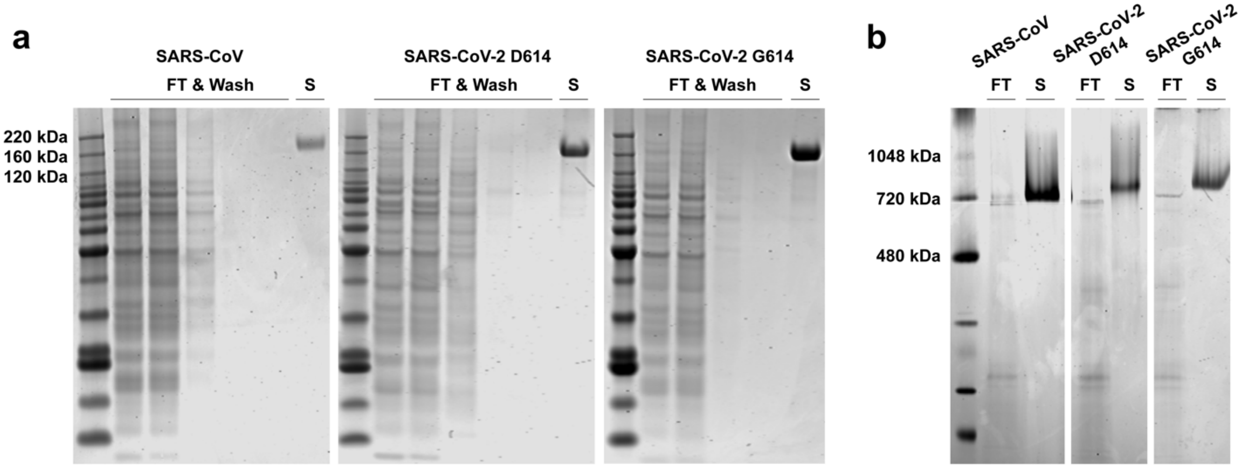

c

DLS	Day 1		Day 2	
	Radius (nm)	%PD	Radius (nm)	%PD
SARS-CoV	9.053	15.1	9.052	15.1
SARS-CoV -2 D614	9.741	14.7	9.519	15.2
SARS-CoV -2 G614	8.690	13.6	8.734	13.9

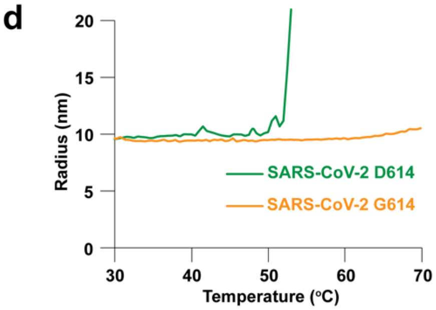

Figure S1. Characterization on purified S. **(a)** SDS-PAGE on three S proteins. Molecular weight ~180 kDa with glycosylation. FT: flow-through. **(b)** Native-PAGE on three S trimers. Apparent molecular weight on Native-PAGE ~720 kDa with glycosylation. **(c)** DLS measurements of three S trimers after SEC purification. Radius around 9 nm with less than 20% polydispersity confirms homogenous S trimers. **(d)** Melting temperatures (T_m) of SARS-CoV-2 S D614 and G614 trimers. G614 trimer shows ~9 degrees higher in T_m than D614 trimer.

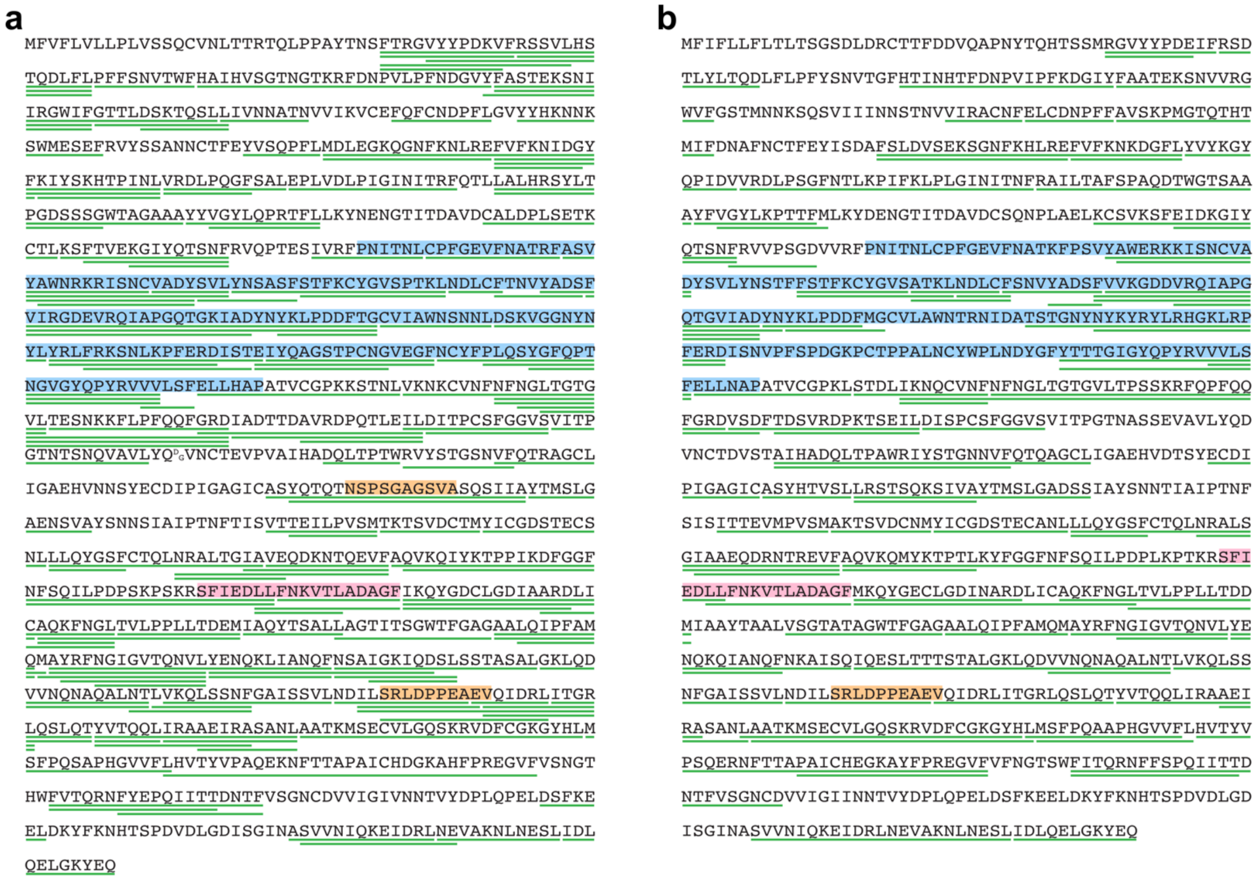

Figure S2. Peptides coverage from pepsin digestion on **(a)** SARS-CoV-2 S and **(b)** SARS-CoV S. Blue shades the RBD region; Orange shades stabilizing mutations over S1/S2 region (SGAG) and S2 central helical apex (PP); Pink shades fusion peptide region.

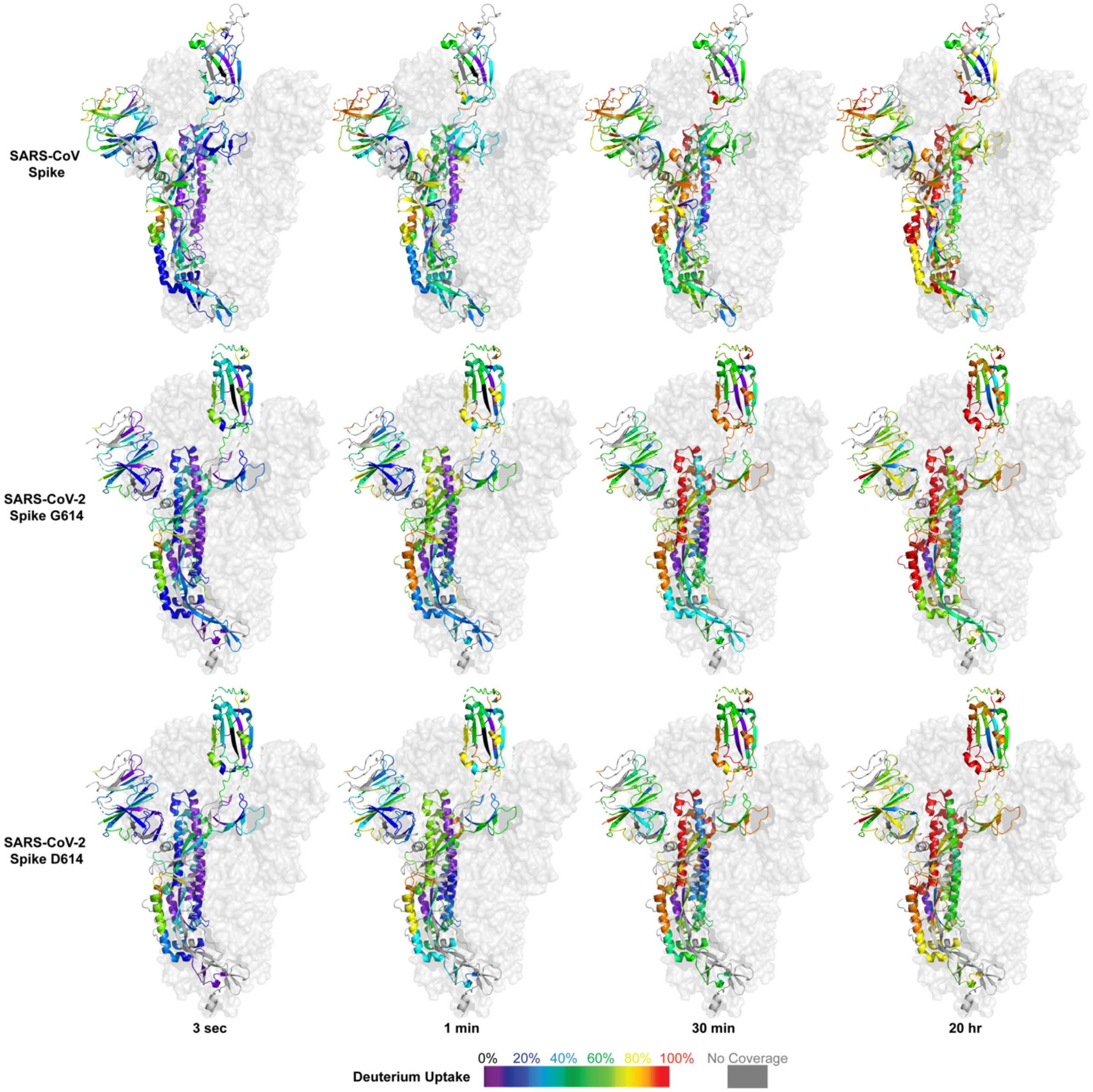

Figure S3. HDX-MS analysis on *apo* spikes, represented as heatmaps, gives the scopes of deuterium exchange levels over incubation time from 3 seconds to 20 hours. PDB: 6CRZ for SARS-CoV S trimer; 6VSB for SARS-CoV-2 S trimer.

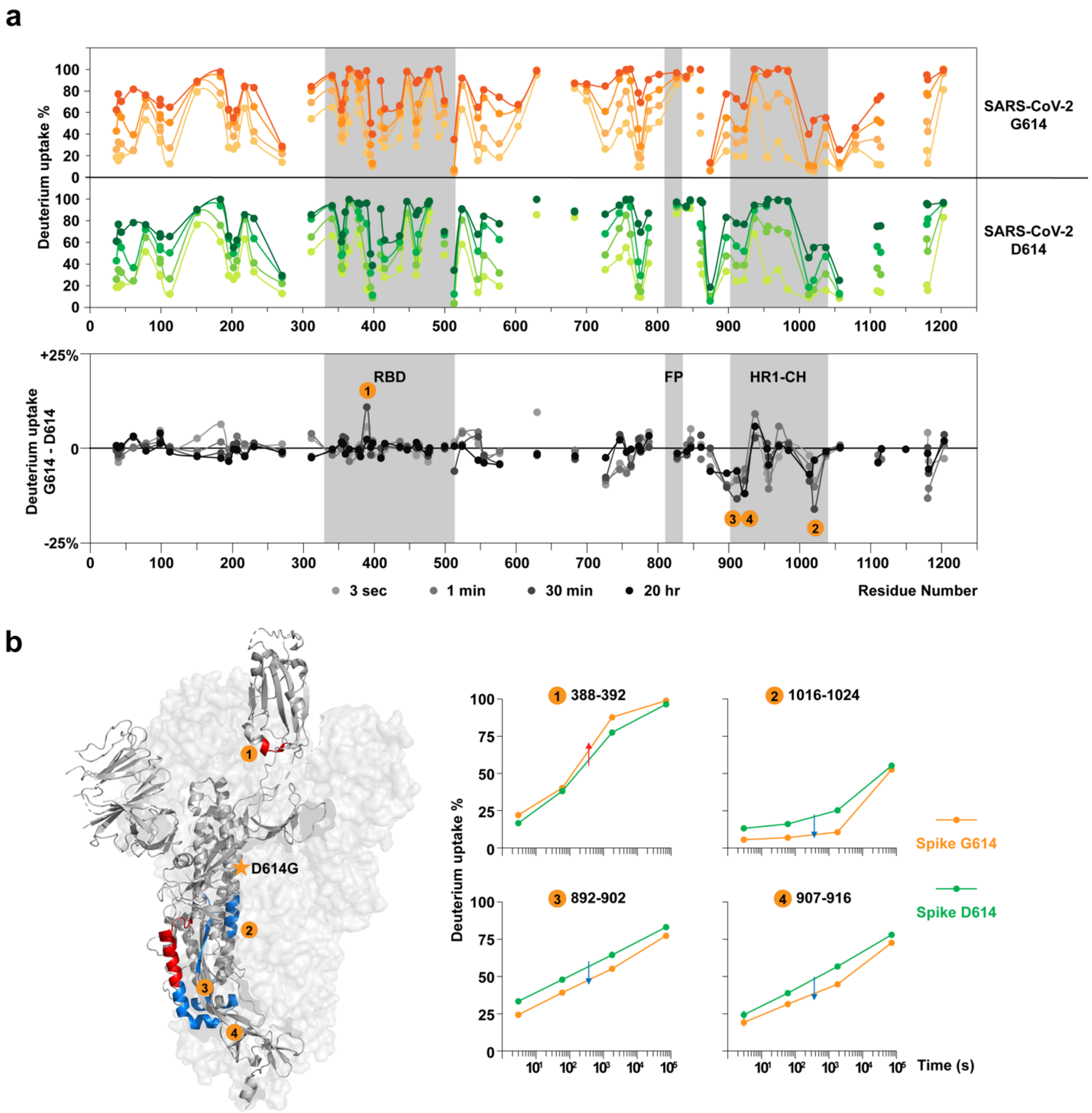

Figure S4. Dynamic differences between SARS-CoV-2 S D614 and G614 trimers. **(a)** Butterfly plots and differential plots of SARS-CoV-2 S D614 vs. G614 reveal the similarities and differences in dynamic behavior across the spike sequences. Grey shadings highlight the RBD, fusion peptide and HR1-CH region. **(b)** Differential heatmaps and uptake plots for peptides showing prominent differences in G614 compared to D614 spikes. In the ribbon diagram heat map, red indicates more exposure and blue indicates more protection in G614 spike vs. D614 spike. G614 spike samples more RBD-up conformation with more exposed RBD C-terminal peptide #1 and exhibits a more stabilized S2 subunit than D614 S as reflected by peptides #2-4.

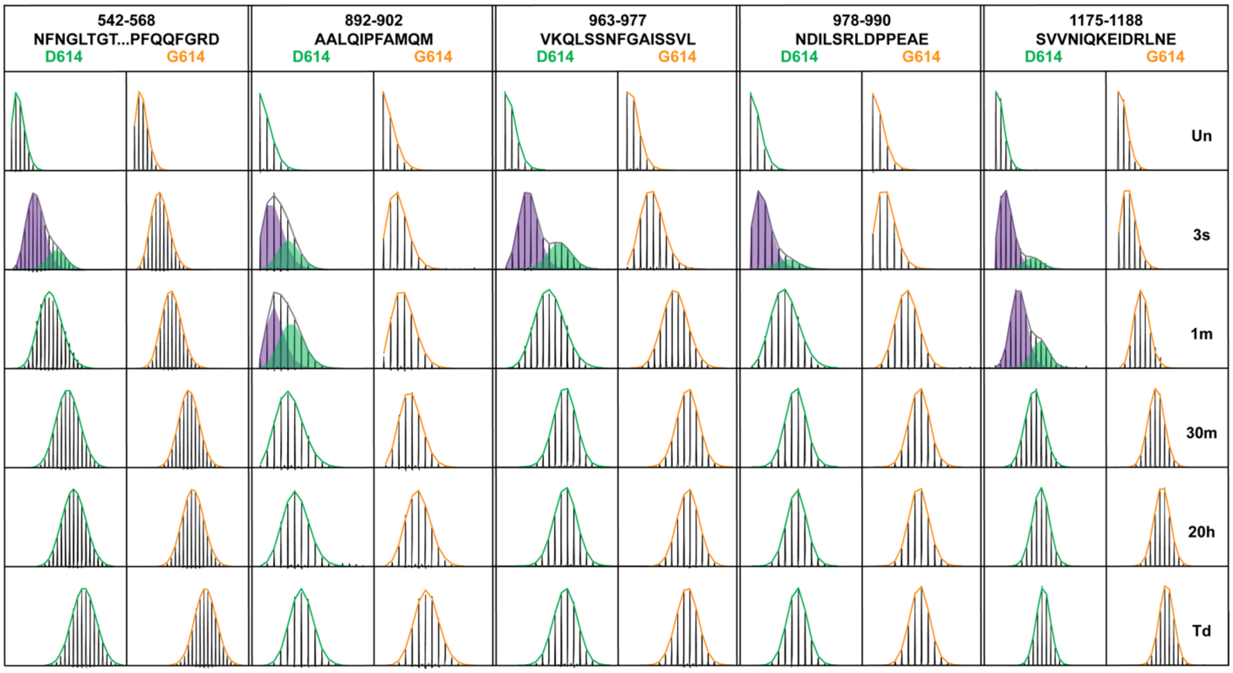

Figure S5. Bimodal m/z spectra show the different dynamic samplings for some peptides in SARS-CoV-2 S D614 compared to the G614 control. Un: undeuterated control; Td: totally deuterated control.

Table S1. Statistics (mean, standard deviation, effect size and p-value) reporting peptides shown in **Figure 3a** indicate significant uptake difference in unbound and hACE2 bound states.

	Unbound (%)	hACE2 Bound (%)	Effect Size (%)	p-value
aa. 472-486				
3 sec	91.45±0.75	69.72±1.14	-21.73	0.0020
30 sec	95.91±0.08	84.90±1.10	-11.01	0.0049
3 min	94.88±0.73	89.74±0.37	-5.14	0.0125
15 min	95.28±0.34	93.46±0.56	-1.83	0.0586
aa. 442-452				
3 sec	82.41±1.52	64.61±0.12	-17.80	0.0037
30 sec	85.69±0.01	72.21±0.96	-13.48	0.0025
3 min	89.53±1.40	87.33±3.94	-2.20	0.5348
15 min	93.50±3.13	91.19±0.79	-2.31	0.4192

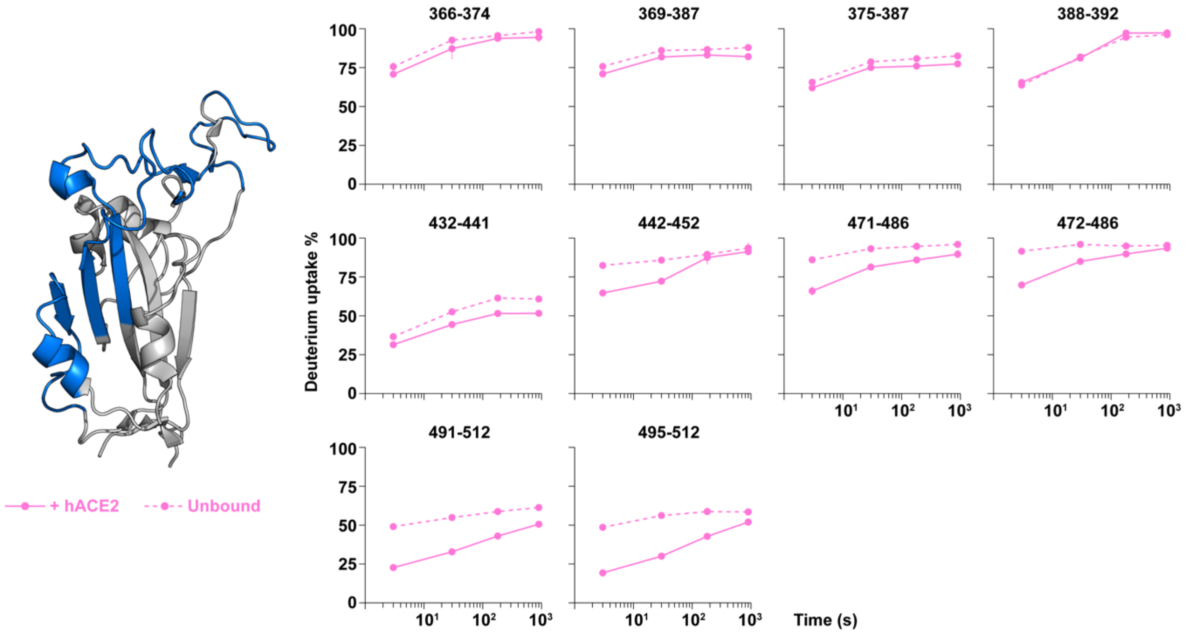

Figure S6. Deuterium uptake plots of the SARS-CoV-2 trimeric tethered RBD peptides showing difference between *apo* and hACE2 bound states. Overlapping plots at peptide 388-392 indicate all RBDs are in the up conformation in both *apo* and hACE2 bound states. PDB: 6W41.

Table S2. Statistics (mean, standard deviation, effect size and p-value) reporting peptides shown in **Figure 3b** indicate significant uptake difference in unbound and hACE2 bound states.

	Unbound (%)	hACE2 Bound (%)	Effect Size (%)	p-value
aa. 472-486				
3 sec	89.35±1.59	69.98±1.67	-19.37	0.0070
30 sec	95.93±1.68	88.23±1.02	-7.71	0.0311
3 min	97.53±0.33	92.87±0.67	-4.66	0.0127
15 min	96.88±0.23	95.90±1.18	-0.98	0.3700
aa. 442-452				
3 sec	82.38±0.69	64.58±1.57	-17.80	0.0046
30 sec	85.65±0.56	73.18±1.13	-12.48	0.0051
3 min	90.48±0.35	85.76±0.60	-4.72	0.0107
15 min	97.14±0.39	92.40±0.50	-4.73	0.0088
aa. 388-392				
3 sec	21.59±0.58	33.35±0.49	11.75	0.0021
30 sec	37.54±0.15	59.91±1.69	22.37	0.0029
3 min	55.61±1.03	81.94±3.71	26.33	0.0105
15 min	75.85±0.18	91.41±1.59	15.56	0.0053
aa. 982-996				
3 sec	19.86±2.18	30.12±0.21	10.26	0.0220
30 sec	36.63±0.63	66.53±0.28	29.91	0.0003
3 min	64.60±0.01	79.80±0.83	15.20	0.0015
15 min	79.90±0.25	88.24±1.54	8.34	0.0171
aa. 542-552				
3 sec	14.23±0.65	19.71±0.34	5.47	0.0088
30 sec	21.80±0.32	33.01±0.24	11.21	0.0006
3 min	36.42±0.58	44.23±0.87	7.81	0.0089
15 min	48.21±0.22	52.48±0.30	4.27	0.0038
aa. 963-977				
3 sec	30.52±0.12	55.27±3.05	24.75	0.0075
30 sec	59.67±0.10	90.29±1.16	30.61	0.0007
3 min	91.11±0.29	95.23±0.66	4.12	0.0149
15 min	98.58±0.80	97.29±0.13	-1.29	0.1552

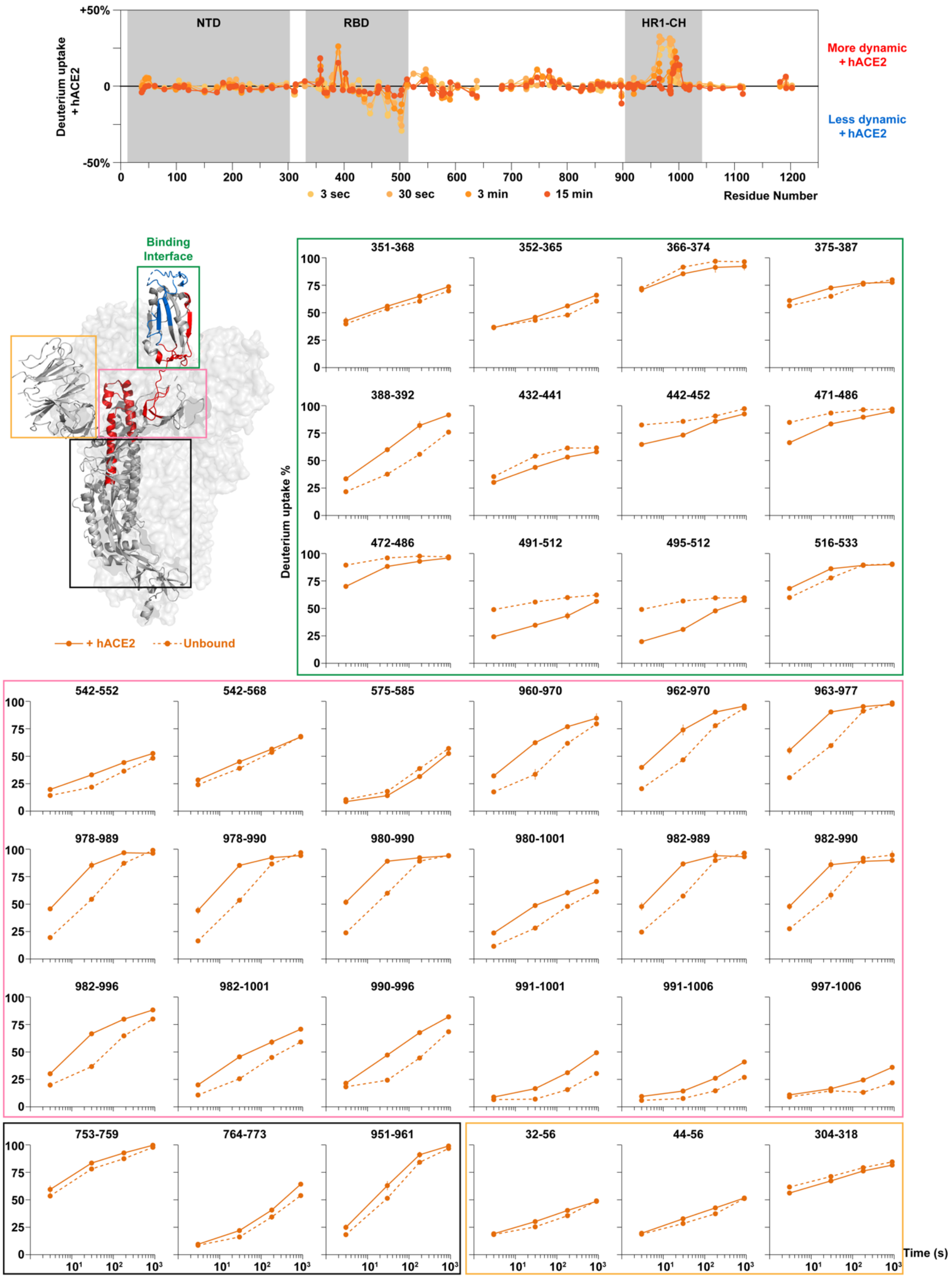
+50%
0
-50%
Deuterium uptake + hACE2
NTD
RBD
HR1-CH
More dynamic + hACE2
Less dynamic + hACE2
0
100
200
300
400
500
600
700
800
900
1000
1100
1200
3 sec
30 sec
3 min
15 min
Residue Number
Binding Interface
+ hACE2
Unbound
Deuterium uptake %
351-368
352-365
366-374
375-387
388-392
432-441
442-452
471-486
472-486
491-512
495-512
516-533
542-552
542-568
575-585
960-970
962-970
963-977
978-989
978-990
980-990
980-1001
982-989
982-990
982-996
982-1001
990-996
991-1001
991-1006
997-1006
753-759
764-773
951-961
32-56
44-56
304-318
Time (s)

Figure S7. Differential plots and deuterium uptake plots of the SARS-CoV-2 S G614 peptides showing difference between *apo* and hACE2 bound states. Green box: RBD; Pink box: S2 central helical apex and hinge region; Black box: bottom S2; Orange box: NTD. PDB: 6VSB.

Table S3. Statistics (mean, standard deviation, effect size and p-value) reporting peptides shown in **Figure 4a** indicate significant uptake difference in unbound and hACE2 bound states.

	Unbound (%)	hACE2 Bound (%)	Effect Size (%)	p-value
aa. 484-500				
3 sec	48.61±0.29	29.75±0.05	-18.85	0.0001
30 sec	50.34±0.53	44.24±0.63	-6.09	0.0091
3 min	50.19±0.29	50.83±0.36	0.64	0.1889
15 min	50.22±0.25	50.98±0.03	0.75	0.0515
aa. 375-379				
3 sec	15.90±0.46	21.44±1.23	5.54	0.0271
30 sec	34.08±0.65	41.46±0.52	7.38	0.0063
3 min	47.89±0.12	57.08±4.50	9.19	0.1019
15 min	63.75±0.65	68.65±1.39	4.90	0.0454
aa. 528-554				
3 sec	19.14±0.56	22.35±1.00	3.21	0.0584
30 sec	31.45±0.41	35.39±0.05	3.94	0.0054
3 min	42.31±0.07	47.33±0.21	5.02	0.0010
15 min	55.90±0.51	59.99±0.24	4.09	0.0095
aa. 960-972				
3 sec	13.57±1.73	25.82±0.61	12.25	0.0111
30 sec	36.19±1.55	51.58±4.91	15.39	0.0517
3 min	67.81±0.23	82.54±0.56	14.74	0.0009
15 min	87.16±1.31	95.03±0.79	7.87	0.0183
aa. 933-943				
3 sec	9.28±1.01	11.70±0.59	2.42	0.0988
30 sec	32.15±3.22	41.38±1.18	9.24	0.0626
3 min	63.83±0.67	73.39±1.02	9.55	0.0080
15 min	84.72±1.49	92.92±0.49	8.21	0.0178
aa. 944-959				
3 sec	23.31±0.37	27.54±2.89	4.23	0.1766
30 sec	38.29±0.09	58.99±2.40	20.70	0.0066
3 min	72.49±3.19	89.72±0.98	17.23	0.0183
15 min	97.50±0.05	102.00±1.24	4.50	0.0361

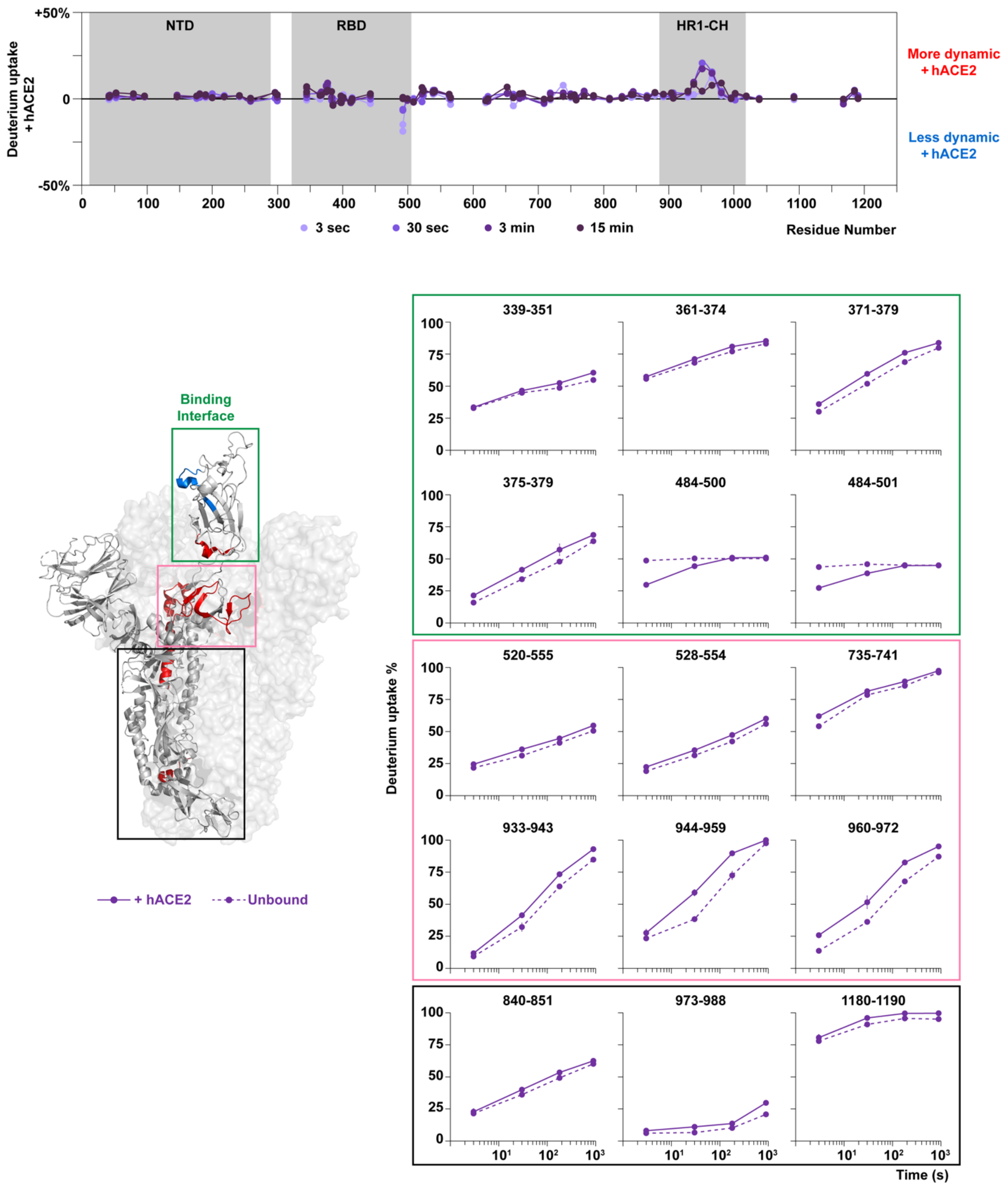

Figure S8. Differential plots and deuterium uptake plots of the SARS-CoV S peptides showing difference between *apo* and hACE2 bound states. Green box: RBD; Pink box: S2 central helical apex and hinge region; Black box: bottom S2. RBD: 6CRZ.

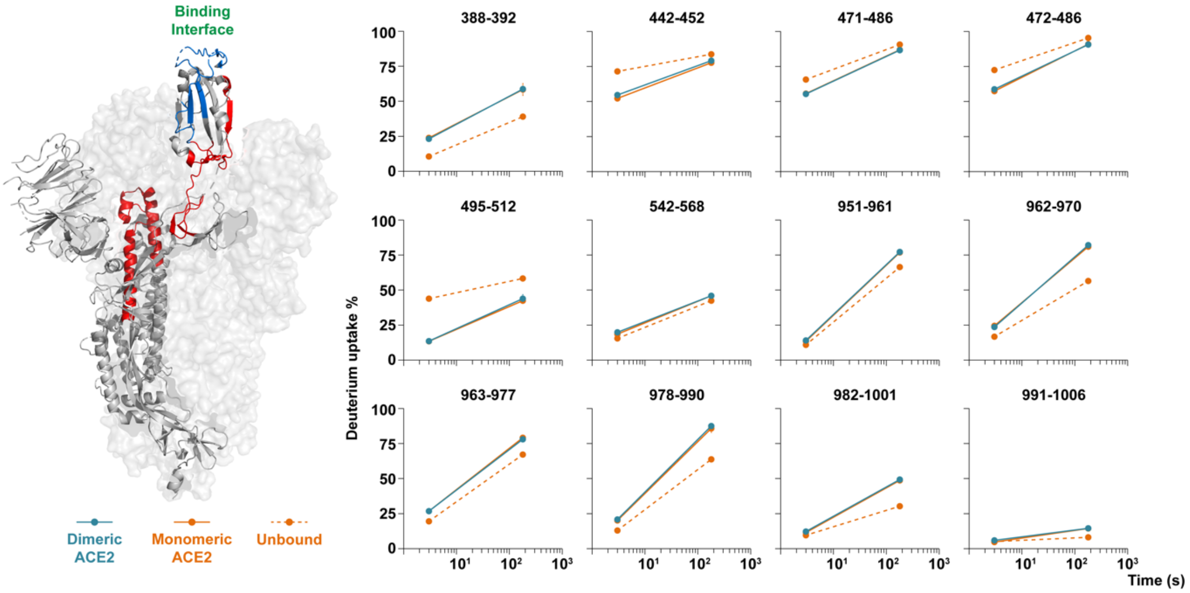

Figure S9. Deuterium uptake plots of the SARS-CoV-2 S G614 peptides showing impacts on hACE2 binding between monomeric and dimeric hACE2. Overlapping points at 3-second and 3-minute incubation indicate dynamic impacts from monomeric hACE2 resemble native-like dimeric hACE2. PDB: 6VSB.

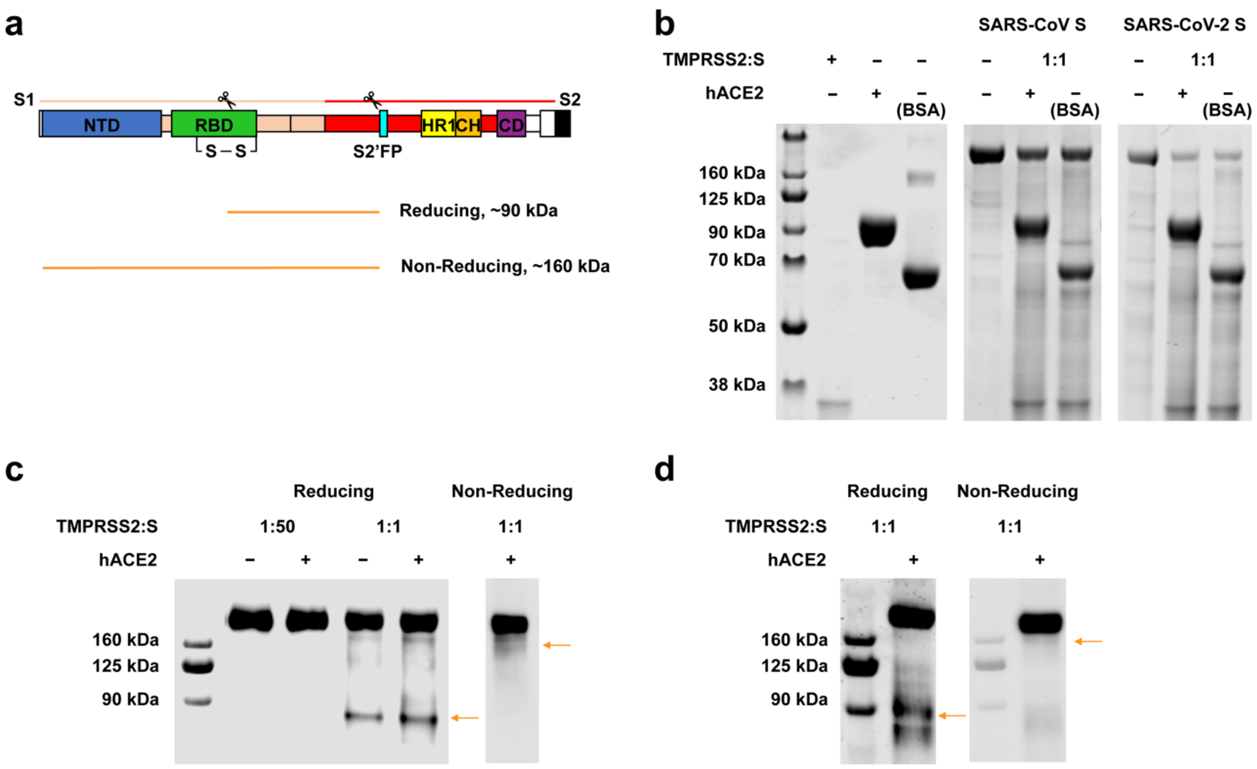

Figure S10. SDS-PAGE and western blot images of soluble TMPRSS2 digested S. **(a)** Suggested TMPRSS2 cleavage sites and possible digested products in reducing and non-reducing conditions. **(b)** SDS-PAGE images of TMPRSS2 digested SARS-CoV and SARS-CoV-2 S with hACE2 or BSA pre-incubation. **(c)** Western blot on TMPRSS2 digested S using primary anti-RBD pAbs. **(d)** Western blot on TMPRSS2 digested S using primary anti-S2 pAbs. Orange arrows indicate the target band shifts due to the reduction of the disulfide bonds shown in (a).
